# Editorial: Stress neurobiology in COVID-19: diagnosis, neuroimaging and therapeutic tools

**DOI:** 10.3389/fneur.2023.1309043

**Published:** 2023-10-30

**Authors:** Alessandro Ferretti, Pasquale Parisi, Pasquale Striano, Alberto Spalice, Paola Iannetti

**Affiliations:** ^1^Pediatrics Unit, Department of Neuroscience, Mental Health and Sense Organs (NESMOS), Faculty of Medicine and Psychology, Sapienza University of Rome, Rome, Italy; ^2^Pediatric Neurology and Muscular Diseases Unit, IRCCS Istituto Giannina Gaslini, Genoa, Italy; ^3^Department of Neurosciences, Rehabilitation, Ophthalmology, Genetics, Maternal and Child Health, University of Genoa, Genoa, Italy; ^4^Department of Maternal, Infantile, and Urological Sciences, Faculty of Medicine and Dentistry, Sapienza University of Rome, Rome, Italy

**Keywords:** COVID-19, SARS-CoV-2, children, neurological complications, pandemic, central nervous system diseases, peripheral nervous system diseases, neuropsychiatric disorder

Coronavirus disease 2019 (COVID-19), caused by severe acute respiratory syndrome coronavirus-2 (SARS-CoV-2), is often asymptomatic in children (1). However, neurological involvement can occur, presenting with a broad range of manifestations (2). The neurological consequences of SARS-CoV-2 can vary from non-specific neurological symptoms to specific central or peripheral nervous system (CNS and PNS) diseases (3). Furthermore, the pandemic has disrupted the care for children with neurological conditions (4, 5) and has also resulted in significant psychological stress, which is still far from being fully quantified (6).

Experimental and clinical data suggest several mechanisms, including the following, that contribute to the neurological manifestations of COVID-19 (7): (1) Direct viral invasion: SARS-CoV-2 can directly invade the CNS by binding to the angiotensin-converting enzyme (ACE) 2 receptor. This receptor is co-expressed with the protease transmembrane serine protease 2 (TMPRSS2) in components of the blood-brain barrier (BBB), glial cells and neurons. This invasion can lead to inflammation when the BBB is compromised. Additionally, the virus may access the CNS via transsynaptic viral passage along cranial nerves V, VII, IX, and X, using the nasopharyngeal, respiratory, and gastrointestinal tracts as entry points. (2) Vascular insufficiency: loss of ACE2 activity caused by SARS-CoV-2 infection could also play a significant role in causing cerebral vascular insufficiency, occlusion and endotheliopathy. (3) Immune system dysregulation: dysregulation of the immune system, leading to a “cytokine storm” and macrophage activation, may result from inefficient innate immunity and impaired viral clearance. This can produce neurologic manifestations through systemic effects and BBB disruption. (4) Autoimmunity: shared sequence similarity between SARS-CoV-2 and sialic acid residues on neural tissue supports an autoimmune hypothesis for post-acute infection neurologic manifestations of COVID-19. Inflammatory responses, direct viral effects on oligodendrocytes, and cerebrovascular abnormalities may precipitate brain demyelination, thereby exacerbating impairment of the central nervous system (8). Lastly, during the COVID-19 pandemic, there was significant exposure to stressful factors, leading to the activation of the hypothalamus-pituitary-adrenal axis and the sympathetic-adreno-medullary axis. In affected individuals, this resulted in increased release of glucocorticoids and adrenaline, leading to impairment of the cardiovascular and immune systems (9).

In this Research Topic, an extensive exploration of the wide-ranging pediatric clinical implications of various pathogenic mechanisms associated with SARS-CoV-2 on both the CNS and PNS, in acute and post-acute phases, is conducted. Possible pathogenesis, laboratory and neuroimaging assessments, treatment responses, and prognosis are identified within each diagnostic framework. Additionally, the Research Topic seeks to shed light on the less explored, non-infection-related, neurological, and psychiatric consequences of the pandemic in the youth.

Casabianca et al. categorize neurological etiopathologies into those determining acute phase issues and post-acute complications. The review highlights the lower comorbidity rates in children compared to adults, leading to reduced pro-inflammatory states and a stronger host immune response. It provides a comprehensive overview of acute and post-acute CNS and PNS complications, emphasizing differences in laboratory and imaging findings compared to other pathogens. Corticosteroids and immunomodulatory therapies are recognized as preferred treatments. Lin et al. present an observational study on acute necrotizing encephalopathy as a SARS-CoV-2-related CNS complication. Fever, impaired consciousness, and seizures should alert clinicians to this condition. Neuroimaging is crucial for diagnosis. Cytokine levels didn't correlate with infection severity. The prognosis is severe, and timely administration of mannitol appeared to reduce the high mortality rate. Cautilli et al.'s case report highlights the CNS involvement post-infection in a young boy who developed acute disseminated encephalomyelitis following an asymptomatic infection. Neurological symptoms guided diagnostic neuro-imaging. High-dose steroids and intravenous immunoglobulin had limited benefit, but subsequent treatments with plasma exchange therapy and rituximab showed some improvement. Unfortunately, the prognosis was unfavorable, resulting in a neurogenic bladder and paraplegia. Perilli et al.'s review focuses on the PNS in SARS-CoV-2 infection. It considers the involvement of cranial nerves (I, II, III, VI, VII, VIII) and reports neuroimaging findings, treatments, and prognoses. The study also overviews cranial nerve palsy in multisystem inflammatory syndrome in children (MIS-C) and the Guillain-Barré syndrome spectrum related to SARS-CoV-2. The authors highlight that microbiological tests frequently yield negative results when neurological symptoms manifest, and serological tests are not consistently conducted. This observation serves as a cautionary note for clinicians. Berloffa et al. described a cohort of children and adolescents with pediatric acute-onset neuropsychiatric syndrome triggered by SARS-CoV-2 infection. They hypothesize a CNS neurochemical imbalance leading to neuroinflammation. To substantiate this pathogenic hypothesis empirically, they have presented evidence suggesting that steroid treatment during the acute phase may offer potential benefits and be well-tolerated. The next two articles in the special issue explore non-infection-related complications arising from the pandemic, specifically focusing on the neurological and psychiatric consequences affecting children and adolescents. Child psychiatry consultations for suicidal attempts significantly increased after the COVID-19 pandemic, as reported by Apicella et al. On the other hand, Bonuccelli et al. observed diverse effects of the pandemic and lockdown on children and adolescents with headaches. It shows how the lockdown altered the lifestyles of youth, resulting in reduced physical activity and increased use of video terminals in most of the interviewed individuals. Finally, Wu et al., by demonstrating that wearing a KN95 face mask can lead to short-term changes in human resting brain function, the consequences of which are not yet known, open an intriguing discussion on what might be the most effective method of preventing neurological consequences in the era of the pandemic.

In conclusion, this Research Topic provides an overview of the neurobiological implications of stress-induced during or after SARS-CoV-2 infection on both the CNS and PNS in pediatric populations. Additionally, it offers insights into the psychiatric and neurological consequences of the COVID-19 pandemic. Special attention has been given to clinical and radiological diagnostic aspects, along with the identification of new therapeutic targets, while also characterizing the prognosis.

Considering the neurological pathologies associated with SARS-CoV-2 infection, some of which are life-threatening, research on recommendations for optimal prevention and treatment in minors is strongly encouraged to limit the spread of infection and, consequently, the potential neurological complications and stress they may cause in youth.

## Author contributions

AF: Conceptualization, Methodology, Writing—original draft, Writing—review & editing. PP: Writing—review & editing. PS: Writing—review & editing. AS: Writing—review & editing. PI: Conceptualization, Supervision, Validation, Writing—review & editing.
